# Methylation of Hypothalamic Tsc1-mTOR Signaling in Regulation of Obesity and Obesity Resistance

**DOI:** 10.1155/2020/8723869

**Published:** 2020-12-30

**Authors:** Yanli Wang, Sijun Diao, Maoqing Hu, Lin Zhang

**Affiliations:** ^1^Department of Endocrinology and Metabolism, Zigong First People's Hospital, Sichuan Province, China; ^2^Chengdu University of Traditional Chinese Medicine, Sichuan Province, China; ^3^Department of Endocrinology and Metabolism, The Third Affiliated Hospital of Chengdu University of Traditional Chinese Medicine, Diabetes Mellitus Prevention and Control Center of Sichuan Province, Sichuan Province, China; ^4^Division of General Medicine, West China Hospital of Sichuan University, China

## Abstract

The Tsc1-mTOR signaling pathway is often related to obesity, and epigenetic modification may lead to expression changes of obesity-related gene. Therefore, we aim to investigate the methylation of the Tsc1-mTOR signaling pathway in regulation of obesity susceptibility. Wistar rats were fed a normal diet or a high-fat diet to develop animal models. Protein and mRNA expression levels of Tsc1-mTOR signaling in the hypothalamus were determined by Western blot and quantitative real-time PCR. Methylation of Tsc1 gene promoter was detected by bisulfite genomic sequence. Both mRNA and protein expression levels of Tsc1 in DIO group hypothalamus were lower; mTOR and its downstream targets S6K1, 4EBP1, and S6 protein expression levels were higher than those of the DIO-R group and the chow group. The Tsc1 gene promoter methylation rate in the hypothalamus was 92.05 ± 3.07% in the DIO group, 87.27 ± 1.91% in the DIO-R group, and 88.18% ± 3.20% in the chow group, respectively, with significantly higher levels in the DIO group. Both the expression levels of Tsc1 gene promoter methylation and Tsc1-mTOR signaling pathway in the hypothalamus of DIO rats and DIO-R rats are different. These findings may shed light on the potential mechanism for the differentiation of obesity susceptibility.

## 1. Introduction

Obesity is known to be the imbalance of energy intake and consumption caused by genetic and environmental factors. In 2016, more than 1.9 billion adults were overweight; of these, over 650 million were obese, and over 340 million children and adolescents aged 5–19 were overweight or obese. In 2019, 38 million children under the age of 5 were overweight or obese [1]. Obesity has become an international public health problem that affects the quality of life, increases the risk of diseases, and aggravates the cost of medical care. Effective weight control can improve obesity complications and coexistence diseases, reducing the total cause of mortality [2]. Therefore, a more comprehensive way to reveal the pathogenesis of obesity and to find safe and effective therapeutic targets has become an urgent medical and social problem for people with obesity. Not all people or rodents will be obese under the same genetic and environmental effects; human and animals have different susceptibility to obesity, and there are obesity and obesity resistance [3]. We will study the pathogenesis of obesity from the perspective of obesity susceptibility.

The mammalian target of rapamycin (mTOR) is involved in cell growth related to protein translation, ribosome biosynthesis, and apoptosis. In the hypothalamus, mTOR regulates body intake and energy perception [4]. 48 hours after fasting, the expression of phosphorylated mTOR and its downstream target ribosome S6 protein kinase 1 (S6K1) in the hypothalamic arcuate nucleus was significantly decreased, while mTOR was activated when eating again. After injecting leucine into the hypothalamic arcuate nucleus, the hypothalamic mTOR activity decreases, and the rats have loss of appetite and lose weight [5]. When the mTOR inhibitor rapamycin was utilized, the above effects disappeared [5]. Tuberous sclerosis syndrome 1 (Tsc1) is a tumor suppressor, and its main function is to inhibit mTOR [6]. Hypothalamic mTOR in Tsc1 knockout rats was activated; the appetite of rats increased and obesity occurred [7]. Therefore, we speculate that hypothalamic Tsc1 may regulate the feeding circuit via the mTOR signaling pathway, addressing the great significance to study the hypothalamic Tsc1-mTOR signaling pathway for better understanding the pathogenesis of obesity.

Epigenetics refers to the genetic transformation of gene expression in body tissues through mitosis or meiosis, with no change in DNA sequence [8]. In recent years, more and more attention has been paid to epigenetic modification in the pathogenesis of obesity. Drastic changes in the environment and lifestyle will lead to changes in the epigenetic modification of DNA in body cells, resulting in the disorders of energy metabolism, which is an important reason for the rapid increase of obesity [9, 10]. A study shows that the hypermethylation of serotonin transporter gene promoter in blood leucocytes is associated with increased BMI and waist circumference [11]. Nutrients can change the expression of key genes related to obesity through DNA methylation [12]; the leptin gene promoter methylation level changes correspondingly when the rats are given different diets [13]. Therefore, from the view of epigenetics, revealing the obesity pathogenesis can bring new treatments for obesity.

In this study, we detected the expression levels of Tsc1 gene promoter methylation and Tsc1-mTOR signaling pathway in the hypothalamus between diet-induced obesity (DIO) rats and diet-induced obesity resistance (DIO-R) rats to investigate the potential regulation mechanism for the differentiation of obesity susceptibility.

## 2. Material and Methods

### 2.1. Animal Models

Animal experiments were conducted in accordance with the Declaration of Helsinki and Chengdu University of Traditional Chinese Medicine Guide for the Care and Use of Laboratory Animals. Male Wistar rats (55–65 g) aged 3 weeks were provided by the Chengdu Dashuo Biotechnology Company, Ltd., with an animal production license number SCXK (Sichuan) 2013-24.

Rats were reared separately at constant temperature (22 ± 2°C) and humidity (50–60%) with 12 h light and 12 h dark cycles. After being acclimatized to the housing for one week, a total of 56 rats were randomly divided into the chow group (*n* = 6) fed with normal feeds and the HFD group (*n* = 50) fed with high-fat feeds. Both diets were purchased from the Chengdu Dashuo Biotechnology Company, Ltd., and the constituent components of the HFD are expressed as follows: 60% normal diet, 10% lard, 15% white sugar, and 15% egg yolk powder (492.8 kcal/100 g) [14]. After being fed for 14 weeks, rats in the HFD group whose body weight exceeded the maximum weight of the chow group were the DIO group, and those less than the average weight of the chow group were the DIO-R group [15]; the other rats were discarded. Epididymal adipose tissue (EAT) and perirenal adipose tissue (PAT) were collected and weighed, respectively.

### 2.2. Quantitative Real-Time PCR

Total RNA from rat hypothalamus was isolated (TRIzol methods), and equal amounts of RNA were reverse transcribed using a mixture of oligodT and random hexamer primers (cDNA Synthesis Kit, K1622, Thermo). Quantitative real-time PCR was performed using 2x Taq PCR Master Mix (Roche) and QuantStudio™ 6 Flex (Applied Biosystems). Expression levels for each gene of interest were normalized to the mean cycle number using real-time PCR for the housekeeping gene encoding *β*-actin. 1 *μ*l of first-strand cDNA product was used for amplification in 9 *μ*l of reaction solution, containing 5 *μ*l of 2x Taq PCR Master Mix and 10 *μ*M of each primer. The following PCR program was used: 95°C for 10 min, followed by 45 amplification cycles of 95°C for 15 s and 60°C for 60 s. Levels of mRNA expression were presented in the form of 2^-*ΔΔ*Ct^ after being normalized to *β*-actin. All experiments were conducted in triplicates.

### 2.3. Western Blot Analysis

Protein extraction reagents were used to extract protein and determine protein concentration. Each protein sample was subjected to sodium dodecyl sulphate polyacrylamide gel electrophoresis (SDS-PAGE) and transferred onto polyvinylidene fluoride (PVDF) membrane. The membrane was blocked by phosphate-buffered solution (PBST) (0.27 g of KH_2_PO_4_, 1.42 g of Na_2_HPO_4_, pH 7.4, 8 g of NaCl, 0.2 g of KCl, and 0.1% Tween 20), and then, the protein expression levels were detected by dilutions of the primary antibodies. Membranes were washed in PBST and then incubated with horseradish peroxidase-conjugated secondary antibody. Bound antibodies were visualized containing an enhanced chemiluminescence reagent. Data were expressed as relative density of the protein normalized to glyceraldehyde-3-phosphate dehydrogenase (GAPDH).

### 2.4. Bisulfite Genomic Sequence (BSP)

After extracting DNA by the DNA Extraction Kit (Tiangen DP304), 1.0 *μ*g of DNA from each sample was treated with bisulfite, using a DNA methylation kit (QIAGEN 59826). PCR amplification of the bisulfite-treated DNA was done. Tsc1 promoter primers included F: 5′-ATGTGAT GTGTTTTAGAATAAAATTTG-3′ and R: 5′-AAATAAAAACAATATCACCACAACTAC-3′; the length of amplified product was 508 bp. PCR conditions were as follows: 95°C for 10 min, followed by 35 cycles of 95°C for 30 s, Tm for 30 s, 72°C for 30 s, and finally 72°C for 10 min. PCR purified products were connected to pGM-T vector and transformed into competent cells and then positive clones and extract plasmids were selected. DNA sequencing was done by Shanghai Ying Biotech Co., Ltd. Based on the principle that sodium bisulfite did not convert thymidine to methylation, we determined whether 50-C-phosphate-G-30 (CpG) was methylated and determined its location.

### 2.5. Statistical Analysis

Data was described as mean ± SD. All comparisons were conducted by ANOVA (one-way analysis of variance) and chi-square tests to identify differences among the groups in the SPSS/PC statistical program (Version 10.0 for Windows; SPSS, Chicago, IL, USA). The *P* value less than 0.05 was considered statistically significant.

## 3. Results

### 3.1. Establishment of Animal Models

**Figure 1:**
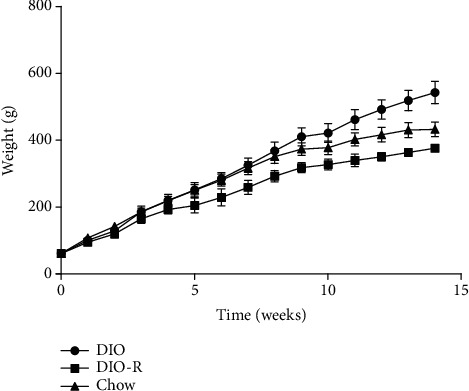
Changes of body weight in the DIO, DIO-R, and chow groups for 14 weeks.

Figure 1 shows the trend of weight gain in three groups. After 14 weeks of feeding, in the HFD group, 8 rats were considered as the DIO group and 6 rats were considered as the DIO-R group. The weight difference between the three groups was statistically significant; the average body weight of the DIO group was 1.44 times higher than that of the DIO-R group. As shown in Table 1, EAT and PAT weights of the DIO group were higher than those of the chow group and the DIO-R group, with statistical significance.

### 3.2. Tsc1 mRNA Levels in the Hypothalamus

**Figure 2:**
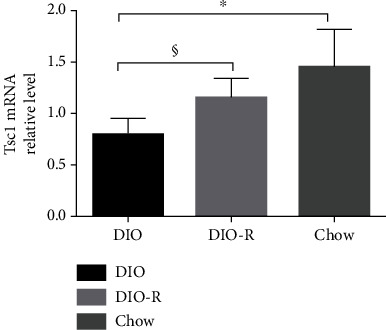
Expression of Tsc1 mRNA in the hypothalamus of each group. ^∗^*P* < 0.001 and ^§^*P* < 0.05 vs. DIO.

As shown in Figure 2, the expression of Tsc1 mRNA in the hypothalamus of the DIO group was lower than that of the DIO-R group (*P* < 0.05) and the chow group (*P* < 0.001). There was no difference between the chow group and the DIO-R group (*P* = 0.05). If the level of Tsc1 mRNA in the DIO group was 100%, the relative level in the DIO-R group and the chow group was 110% and 137%, respectively.

### 3.3. Tsc1-mTOR and Its Downstream Protein Levels in the Hypothalamus

**Figure 3:**
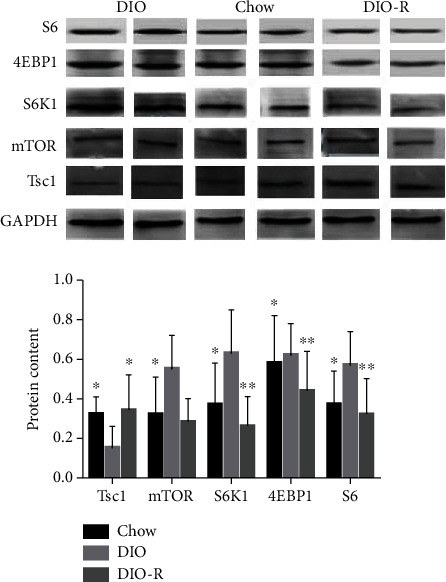
The expression levels of Tsc1, mTOR, S6K1, 4EBP1, and S6 in the hypothalamus of each group. ^∗^*P* < 0.05 and ^∗∗^*P* < 0.001 vs. DIO.

We examined the expression levels of Tsc1 and mTOR and its downstream protein S6K1, 4EBP1, and S6 in the hypothalamus of rats. As shown in Figure 3, compared with the DIO-R group and the chow group, the Tsc1 level in the hypothalamus of the DIO group was relatively downregulated, and the mTOR level was relatively upregulated, which was also observed in S6K1, 4EBP1, and S6 levels; the differences were statistically significant. There were no differences between the DIO-R group and the chow group.

### 3.4. Methylation of Tsc1 Gene Promoter in the Hypothalamus

**Figure 4:**
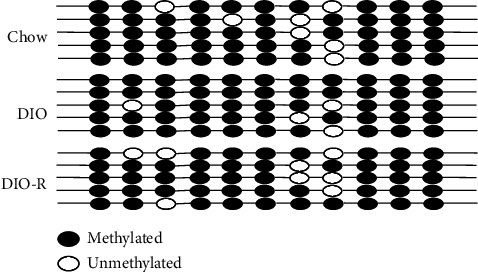
Methylated and unmethylated sites of Tsc1 gene promoter in the hypothalamus of each group.

We detected the methylation level of Tsc1 gene promoter in rat hypothalamus by BSP; the sequenced fragment contained 11 CpG loci. The results showed that the methylation level of Tsc1 gene promoter in the hypothalamus of the DIO group presents an increasing trend. The methylation rate in the DIO, DIO-R, and chow groups was 92.05 ± 3.07%, 87.27 ± 1.91%, and 88.18% ± 3.20%, respectively. The methylation ratio of the Tsc1 gene promoter in the hypothalamus of the three groups was different (*P* < 0.001), the Tsc1 gene promoter methylation level in the DIO group was higher than that of the DIO-R group and the chow group (*P* < 0.017), and there was no difference between the chow group and the DIO-R group (Table 2, Figure 4).

## 4. Discussion

This study demonstrated that rats showed obesity and obesity resistance under the same high-energy diet. DIO rats had similar conditions to human obesity, with increased body fat, while DIO-R rats showed the opposite. Under the same genetic and environmental effects, the susceptibility of rats to obesity is different. Studies have reported that genetic factors can affect 40% difference in energy expenditure [16]; even if given the same diets, the rats with obesity genes are prone to obesity resistance [17]. The occurrence of obesity and obesity resistance is a metabolic process involving multiple systems, and some hormones and signal pathways may be involved in this process. Orexin may promote obesity resistance by increasing spontaneous physical activity and influencing the metabolic state of orexin-responsive hypothalamic neurons [18]. There are substantial differences in the transcriptomes, phenotypes, and metabolic processes between obesity and obesity-resistant mice [19]. Insulin-like growth factor 2 mRNA-binding protein 2/IGF-II mRNA-binding protein 2- (IGF2BP2/IMP2-) deficient mice have a higher level of the uncoupling protein 1 (UCP1) polypeptide in brown fat, contributing to the higher energy expenditure and resistance to diet-induced obesity [20]. These studies are still in their infancy, but the results have far promise in helping to explain the variation in obesity susceptibility.

The mTOR signaling pathway plays an essential role in the regulation of whole-body energy balance in the central nervous system (CNS). mTOR and its downstream target S6K1 are widely expressed in rat hypothalamus; mTORC1 overexpression in the hypothalamus can lead to changes in rats' feeding behavior and obesity occurrence [21]. Overnutrition impairs mTORC1 activity and decreases mTORC1 signaling in rat hypothalamus, which is implicated to assist in the development of hyperphagia, weight gain, and leptin resistance in HFD-induced obesity [22]. Phosphorylated S6K1 is increased in the AgRP neurons, but is suppressed in the ventromedial nucleus of the hypothalamus (VMH) under fasting or in leptin-deficient ob/ob mice [23], and refeeding markedly decreases mTORC1 activity [24]. Tsc1 is a classic suppressor in the upper reaches of mTOR, and mTORC1 signal is activated during knockout of Tsc1 gene in rat POMC neurons; this is not only associated with cell hypertrophy but also reduces the projection of the neurite axon in the paraventricular nucleus of the hypothalamus, which is associated with the occurrence of nutritional obesity [25]. In this experiment, we found that compared with DIO-R rats, the expression levels of Tsc1 mRNA and protein in DIO rat hypothalamus showed a downward trend, and the expression levels of mTOR and its downstream targets S6K1, 4EBP1, and S6 increased. It is possibly suggested that the hypothalamic Tsc1-mTOR signaling pathway plays a regulatory role in the obesity susceptibility of rats. The expression of Tsc1 in DIO rats is downregulated, the mTOR signal in the hypothalamus is activated, and obesity in rats occurred, while DIO rats, on the contrary, showed obesity resistance.

A study evaluated the effects of genetic and environmental factors on the difference in height, weight, and BMI from birth to age 19 [26]. The results indicated that these factors had little effect on the genetic factors at birth. However, with the increase of age, the influence of genetic factors on the difference of body weight and BMI was more than half or even more [27]. The researchers also found that common environmental factors were especially eager for prepuberty girls [28]. How the mechanism of environmental factors impacts the genetic susceptibility is not very clear; some researchers have suggested that epigenetic variation determines the organism's adaptation to the environment. Similarly, the environment is considered to be a factor affecting the expression of susceptible genes [28]. The mTOR pathway acts as an energy receptor to regulate the energy balance and is regulated by a variety of signaling pathways through various factors. The mTOR signaling pathway in the mouse hypothalamus with Tsc1 gene deletion is activated, and the food intake of mice is increased and obesity occurs [7]. Our study suggests that the hypothalamic Tsc1-mTOR signaling pathway may play an important role in obesity susceptibility of rats, but the precise mechanism underlying this phenomenon is not yet clear.

Epigenetic status changes have been verified to be associated with many diseases, including cancer, cardiovascular disease, neurodegenerative diseases, mental disorders, and autoimmune diseases. Numerous animal and human experiments in the early stage suggest that the abnormal methylation level of obesity-related genes may be involved in the occurrence of obesity. Leptin resistance is a relevant factor in the pathogenesis of obesity, and epigenetic modifications could contribute to leptin expression and signaling disturbances in obesity [29]. Dick et al. conducted an analysis of 450 million CpG sites and made an association between BMI with raised DNA methylation at hypoxia-inducible transcription factor 3A (HIF3A) [30]. Colonic DNA methylome was preprogrammed in young obese mice inducing genetic characteristics of being prone to tumor after aging [31]. The relationship between DNA methylation and obesity needs further study.

The methylation status of CpG loci in Tsc1 gene promoter is a determinant to the independence of Tsc1 gene transcription. Therefore, we dare to speculate whether the methylation level of Tsc1 gene promoter is related to the abnormal expression levels of Tsc1-mTOR signaling in the hypothalamus, resulting in different obesity tendencies in rats. In this study, we found that the Tsc1 gene promoter methylation rate in the hypothalamus was higher in the DIO group than in the DIO-R group. The first half studies suggested that the expression levels of Tsc1 mRNA and protein in the hypothalamus of the DIO group were lower than those of the DIO-R group, which is consistent with the difference in methylation level of Tsc1 gene promoter between the two groups. It is suggested that the methylation level of Tsc1 gene promoter in the hypothalamus may be the epigenetic mechanism regulating the differentiation of obesity susceptibility in rats. This finding presents Tsc1 as an important candidate gene for patients with obesity. The abnormal level of CpG methylation in the promoter region plays an important role in the pathophysiology of obesity and provides evidence for the epigenetic cause of obesity.

## 5. Conclusion

Our investigation demonstrates that gene promoter methylation and expression of Tsc1 are closely related to the degree of obesity induced by high-fat diet; hypothalamic Tsc1 serves an important role in the maintenance of homeostasis of energy metabolism. Tsc1 in the arcuate nucleus is reportedly important in the hypothalamus; we did not harvest the mediobasal hypothalamus; the following research will be further improved. In addition, the detection of the mTOR signaling pathway in the hypothalamus is not comprehensive, and further studies are needed to block Tsc1-mTOR signal to confirm our results.

## Figures and Tables

**Table 1:** EAT and PAT weights in three groups (mean ± SD).

Group	*n*	EAT (g)	PAT (g)
Chow	6	4.47 ± 0.74	5.45 ± 0.94
DIO	8	7.51 ± 1.15^§^	10.45 ± 1.67^§^
DIO-R	6	3.13 ± 1.12^∗^^#^	3.88 ± 0.76^∗^^#^

^∗^ *P* < 0.05 and ^§^*P* < 0.001 vs. the chow group; ^#^*P* < 0.001 vs. the DIO group.

**Table 2:** Methylation of Tsc1 promoter in the hypothalamus of three groups.

Group	Methylated	Unmethylated	Total	Methylation ratio
Chow	288^∗^	42	330	88.18% ± 3.20%^∗^
DIO	408	32	440	92.05 ± 3.07%
DIO-R	282^∗^	48	330	87.27 ± 1.91%^∗^

^∗^ *P* < 0.017 vs. DIO.

## Data Availability

Data are available on request.
